# The effects of Ghana's capitation policy on hospital Under-5 mortality in the Ashanti Region

**DOI:** 10.4314/gmj.v56i3.8

**Published:** 2022-09

**Authors:** John K Yambah, Naasegnibe Kuunibe, Roger A Atinga, Kindness Laar

**Affiliations:** 1 University Health Services, Akenten Appiah-Menka University of Skills Training and Entrepreneurial Development, Kumasi, Ghana; 2 Department of Health Policy, Management and Economics, School of Public Health, Kwame Nkrumah University of Science and Technology, Kumasi, Ghana; 3 Department of Economics, Faculty of Social Science and Arts, SD-Dombo University of Business and Integrated Development Studies, Ghana; 4 Department of Public Administration and Health Services Management, University of Ghana Business School, Accra, Ghana

**Keywords:** health insurance, capitation policy, under-5 mortality, Ashanti Region, Ghana

## Abstract

**Objective:**

The study estimated the capitation policy's effect on the under-5 mortality (U5MR) rate in hospitals in Ashanti Region.

**Design:**

We used an interrupted time series design to estimate the impact from secondary data obtained from the DHIMS-2 database. Monthly under-5 deaths and the number of live births per month were extracted and entered into Stata 15.0 for analyses. The U5MR was calculated by dividing the number of live deaths by the number of live births for each of the 60 months of the study.

**Setting:**

Health facilities of the Ashanti Region with Data in the DHIMS 2.

**Intervention:**

the level and trend of U5MR for 31 months during the Capitation Policy implementation (January 2015 to July 2017) were compared with the level and trend 29 months after the withdrawal of the capitation policy (August 2017 to December 2019).

**Outcome measures:**

changes in trend or level of U5MR after the withdrawal of capitation.

**Main Results:**

During the capitation policy, monthly U5MR averaged 10.71 +/-2.71 per 1000 live births. It declined to 0.03 deaths per 1000 live births (p=0.65). After the policy withdrawal, the immediate (increase of 0.01 per 1000live births) and the trend (decline of 0.13 deaths per 1000 live births per month) were still not statistically significant.

**Conclusion:**

We conclude that the capitation policy did not appear to have influenced under-5 mortality in the Ashanti Region. The design of future healthcare payment models should target quality improvement to reduce under-5 mortalities.

**Funding:**

None declared

## Introduction

Despite significant strides made during the millennium development goals, Sub-Saharan Africa still contends with very high under-5 mortality (U5MR). Ghana's U5MR, 52 per 1000 live births^1^, is twice as higher as the Sustainable Development Goal (SDG) target^2^ and is considerably higher than the average of other low and low-middle-income countries (LMICs).^3^ The U5MR in Ghana shows wide regional variations, with the two most populous regions, Greater Accra and Ashanti regions, recording the lowest and highest, respectively.^4^

Nearly half of all U5MR is accounted for by infant mortality^4^, an indicator that some researchers argue reflects health services efficiencies, especially in Africa.^5^,^6^ Other contributory factors of U5MR are linked to malnutrition,^7^,^8^ infectious diseases, seven poverty^9^,^10^ and the quality of child health services, especially immunisation.^11^

In general, an important determinant of health outcomes and mortalities is the health financing regime.^12^,^13^

The public health financing policy, whether of health insurance, fee for service or other health financing arrangements, has implications on vulnerable populations' health outcomes.^13^ Health insurance regimes, for example, have enhanced access to health services and outcomes across populations.^14^–^17^ However, evidence on the link between health outcomes and health insurance payment mechanisms such as capitation is mixed. Most studies in high-income countries showed that capitation improved access to physician care 18 and reduced premature mortality and overall mortality rates.^19^,^20^ In China, health service quality and neonatal mortality did not change significantly after implementing the capitation policy.^21^ In sub-Saharan Africa, studies assessing the impact of capitation policies on health outcomes are rare. We came across a retrospective cohort study that showed that the capitation policy in Ghana reduced hospital visits and resulted in worse outcomes.^22^ However, these outcomes did not include U5MR. The paucity of literature on this important field tends to constrain decision-making regarding the real impact and benefit of capitation to vulnerable populations under-5. Based on the gap in the literature, we drew on robust DHIMS 2 data to explore the impact of the capitation pilot policy in Ghana on U5MR in a regional health system.

Capitation is a payment system whereby health facilities are allocated a fixed sum of money for a defined period according to their relative size and composition of the user population.^12^ Capitation systems are thought to provide incentives for preventive healthcare, better adherence to insurance regulations and efficiency in cost and quality. Collectively, these attributes could improve outcomes, including childhood survival. However, it is recognised that quality health services may decline if capitation is not accompanied by supervision, monitoring and auditing of health facilities because of known adverse effects such as dumping and cream skimming.^23^–^26^

In Sub-Saharan Africa, the effects of capitation policies vary because of design characteristics and implementation challenges. For example, in Kenya, providers were paid a retrospective capitation which providers contend was delayed.^27^ This sharply contrasts with Ghana, which paid capitation prospectively and on time.^28^ The increased focus on capitation-based payment methods, especially in developing countries, as strategies towards the SDGs and UHC (Universal Health Coverage)^13^ require monitoring how these policies contribute towards attaining SDG 3.2, which aims at reducing U5MR to less than 25 per 1000 livebirths.

## Methods

### Study setting and intervention

The Ashanti region has a population of nearly six million and constitutes about 20% of the population of Ghana. Ghana has Overall insurance coverage of 40%, with the Ashanti Region leading in a number of persons with active card renewals and active registrants. Of the about 1759 health facilities in the Region, 25 of these are district hospitals in the 44 administrative areas in the Region. These hospitals receive referrals from public and private lower-tier health facilities (Clinics, Polyclinics and CHPS compound) in their enclave and serve as the gatekeeper for onward care in the only teaching hospital in the region. Hospitals in the region are responsible for both generalised and specialized care, including maternal and child health services, with very complicated cases being referred to the teaching hospital. The health system in Ghana is tiered and begins from CHPS, the basic level, through Health Centres and Polyclinics to the District and Regional hospitals with the Teaching hospitals at the Apex.

Under Ghana's health insurance policy, neonates access free care under a mother's health insurance coverage for up to three months. Beyond this age and up to a year after birth, children are expected to receive free health care. Also, children under 18 years belong to the exempt premium group only required to pay fees for registration provided at least a parent is enrolled with the scheme.

The pilot capitation policy began in 2012 with a specific design mixture of the capitation payments with other payment methods. In this policy, primary outpatient services were to be covered by per capita advanced monthly payments to credentialed facilities selected by beneficiaries. Inpatient care and pharmaceuticals were reimbursed retrospectively by GDRG (Ghana Diagnosis Related Group) and FFS (Fee For Service), respectively using the NHIS approved Tariff list. In 2016 government began to extend the policy to other regions, suggesting, without a study, that the pilot successfully achieved its cost containment objective. However, in August 2017, the policy was withdrawn.

### Study design and source of data

A single group interrupted time series analysis (ITSA) design was employed using monthly routine secondary data. The ITSA design allowed for us to estimate the policy effects of capitation, as well as the immediate and long term impacts of the policy withdrawal.^29^,^30^ Data on under-5 mortality was extracted from DHIMS2 for analysis. We considered 31 months during the policy implementation and 29 months after its withdrawal for this study.

### Outcome variable

The facility's monthly under-5 mortality was converted to the standardised institutional monthly U5MR. This was done by dividing the crude under-5 deaths by the total number of live births recorded in the month. This measure is more useful compared to the monthly counts of under-5 deaths.

### Timing and variables

Time was counted from January 2015 to December 2019. We coded the series from 1–31 (from January 2015 to July 2017) as the period of the capitation policy and 32–60 (August2017-December 2019) as the period after the policy withdrawal. The standardised U5MR was analysed across these periods. For the impact analysis of the capitation policy, we included two dummy variables: series 1–31 for the capitation policy and series 32–60 for the policy withdrawal.

### Statistical analysis

Summary statistics of the mean, standard deviation, and range of U5MR during and after the capitation policy were presented in tables. Ordinary least square regression analysis (segmented regression model) at lag one was employed to analyse U5MR levels and trends during the capitation policy and after its withdrawal. The regression equation is presented in equation (1).

*U*5*t* = *φ*_0_ + *φ*_1_*T_t_* + *φ*_2_*Z_t_* + *φ*_3_*Z_t_T_t_* + *τ_t_*...(1), where *φ*_0_ represents the existing level of under-5 mortality at January 2015; *T_t_* is the time from January 2015 until the end of July 2017 (during the capitation policy), *φ*_1_ is the pre-existing U5MR from January 2015 during the capitation policy, *Z_t_* a dichotomous variable representing the withdrawal of the capitation policy; *φ*_2_ represents the immediate impact of the withdrawal of the policy and measures the change in the U5MR in the month immediately following the withdrawal compared to the pre-existing level (the counterfactual); *Z_t_T_t_* is an interaction term representing the period of the capitation policy withdrawal between August 2017 and December 2019; *φ*_3_ represents the long-term impact of the withdrawal of the capitation policy and measures the difference between pre-and post-withdrawal trends in U5MR. Tau *τ* is the random error term assumed to be normally distributed. We calculated the P-values to determine the level of significance in each of the parameter estimates. We employed the generalised linear model (GLM) to correct autocorrelation and reported the original and transformed Dubin-Watson (DW) statistics. DW Values close to 2 in the original statistic indicate no autocorrelation. Greater or less than 2 DW values represent the presence of autocorrelation. When autocorrelation was present, the transformed DW statistic confirmed the correction resulting from using the GLM model.

Ethical clearance was obtained from the Committee on Human Research Publications and Ethics of the Kwame Nkrumah University of Science and Technology (reference number CHRPE/AP/426/20).

## Results

### Descriptive statistics

Table 1 presents the descriptive statistics as well as the results from the regression model. Specifically, we present the mean, standard deviation, and range of U5MR during the capitation policy and the immediate and long-term impact of its withdrawal on U5MR.

**Table 1:** U5MR during and after capitation

Policy Period	Model Results		Summary statistics			
	Parameter	Estimate	Mean	Std. Dev.	Minimum	Maximum
During capitation	Level (*φ*_0_)	11.03***	10.71	2.47	6.20	16.09
	Trend (*φ*_1_)	-0.03				
After withdrawal	Level change (*φ*_2_)	0.19	10.23	1.36	7.15	13.38
	Trend change (*φ*_3_)	-0.13				
DW statistic	Original	1.72				
	Transformed	2.00				

During the capitation policy, U5MR in hospitals averaged U5MR 10.71 +/- 2.47 per 1000 live births and ranged between 6.20 to 16.09 per 1000 live births. After the withdrawal of the policy, U5MR averaged 10.23 +/- 1.36 per 1000 live births and ranged from 7.15 to 13.38 per 1000 live births.

**Figure 1:**
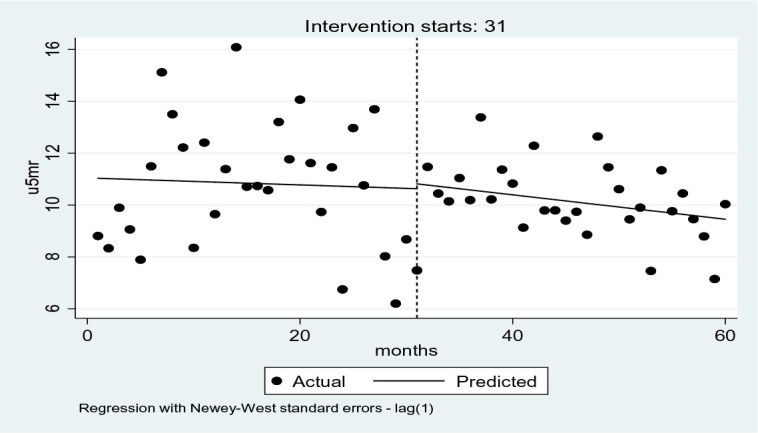
ITSA graphs of U5MR levels and trends during and after capitation

The model results in Table 1 show that in January 2015, U5MR was about 11 deaths per 1000 live births. This declined to 0.03 deaths per 1000 live births, albeit not statistically significant. In the month immediately after the policy withdrawal, an increase of U5MR by 0.10 deaths per 1000 live births was recorded (p=0.94). The trend declined at 0.01 (p=0.87) in the post-capitation policy period. Figure 1 is a graphical presentation of the levels and trends during and after capitation.

## Discussion

This study examined the effect of the capitation payment policy and its subsequent withdrawal in the Ashanti Region of Ghana on hospital U5MR. Our study is the first to evaluate the impact of the capitation payment system on U5MR in Ghana. We used data from DHIMS II and employed the single ITSA to demonstrate further the strength of routine data in evaluating complex health financing systems such as capitation. Aside from the fact that primary data collection is costly, it is usually based on limited data points with short-term follow-up periods, making evaluating longer-term effects impractical. We found that, in line with similar studies on the effects of the NHIS, the capitation policy did not affect U5MR in the Ashanti region of Ghana. Even though we found a declining trend attributable to capitation, this was not statistically significant.

First, the non-significant declines in U5MR suggest no improvements in quality brought about by the capitation. One may infer that in line with earlier studies,^31^,^32^ capitation did not influence the quality of childcare services. On another hand it could mean that the health sector may not have had enough health services human capacity for effective output^33^ to impact on quality and therefore, U5MR. It is also possible that, like in other developing countries, hospitals in Ghana are less equipped to effectively deliver quality care that reduces U5MR significantly even if payment systems are appropriate.^33^ Sec-ond, it is also possible that capitation initially improved access^22^ and resulted in crowding at hospitals, which in turn serves to deter people from visiting hospitals.^22^ Once people do not visit hospitals, due to crowding and delays in receiving care, the likelihood of children receiving less skilled or unskilled care from alternate providers becomes very high. It could then contribute to the non-effect finding. Third, other non-hospital related social determinants, besides capitation, may influence the U5MR more significantly.^34^,^35^ Results from our study lend support to findings of previous studies in Ghana^34^,^35^, that health insurance did not affect under-5 and neonatal mortality. Finally, it is possible that the policy indirectly increased preventive healthcare services.^11^,^36^

This could potentially improve health outcomes as evidenced by the declining albeit non-significant trends of U5MR associated with the capitation policy.

There are some limitations of the study to be acknowledged. The U5MR calculated could be an underestimation because we assumed a negligible number of stillbirth outcomes and the combined numbers of normal and Caesarean deliveries that served as the denominator for this estimate are approximately equal to the number of live births. We also assumed that each birth resulted in a singleton baby. Accurately determining these from the dataset was difficult because the number of multiple deliveries as well as the outcomes may not have been known at the time of their entries into the DHIMS 2 database. We employed the ITSA, which allowed us to observe monthly changes in U5 mortality under the capitation policy, which lasted for a short period. While the data points are plausible for assessing observed changes during the implementation, we acknowledge that a potentially longer period could better shape the capitation impact on clinical outcomes such as mortality. However, this was not possible due to the suspension of the policy. In addition, the ITSA approach did not allow us to account for other possible cultural or socio-economic confounders of U5 mortalities. But by estimating the effect over time, the potential influence of these confounders was minimised. Notwithstanding, we urge caution in the interpretation of the results.

## Conclusion

We found that the capitation policy did not affect U5MR in the Ashanti Region during its implementation.
